# Exploring physicians’ perspectives on influencing factors of chronic non-specific low back pain: a qualitative study

**DOI:** 10.1080/02813432.2026.2640406

**Published:** 2026-03-16

**Authors:** Rob Vanderstraeten, Antoine Fourré, Isaline Demeure, Hilde Bastiaens, Christophe Demoulin, Jozef Michielsen, Sibyl Anthierens, Nathalie Roussel

**Affiliations:** a Faculty of Medicine and Health Sciences (MOVANT), University of Antwerp, Antwerp, Belgium; b Department of Neurosciences, Université de Mons, Mons, Belgium; c Faculty of Medicine and Health Sciences, Family Medicine and Population Health, University of Antwerp, Antwerp, Belgium; d Department of Sport and Rehabilitation Sciences, University of Liege, Liege, Belgium; e Faculty of Motricity Sciences, UCLouvain, Louvain-la-Neuve, Belgium; f Antwerp Surgical Training, Anatomy and Research Centre, University Hospital of Antwerp, Antwerp, Belgium

**Keywords:** Chronic low back pain, qualitative, physician, conceptualisation, biopsychosocial approach

## Abstract

**Purpose:**

This study aimed to assess physicians’ perspective of non-specific chronic low back pain (cLBP), focusing on whether they attribute the condition to single or multiple influencing factors. It also explored how these factors are conceptualised and whether physicians’ expressed attributions align with the biopsychosocial approach advised in the guidelines for the management of cLBP.

**Methods:**

This exploratory qualitative study used a clinical vignette depicting cLBP and employed a deductive framework analysis. Licensed general practitioners and trainees were asked to identify contributing factors to the patient’s cLBP. Five predefined themes, ‘Beliefs’, ‘Previous experiences’, ‘Emotions’, ‘Patients behaviour’ and ‘Contextual factors’, were analysed to assess their framing within a biopsychosocial approach.

**Results:**

Seventy-six physicians participated. They used very brief responses when reporting contributing factors to cLBP (median: 11 words). While 71% identified sedentary behaviour as a contributing factor, only 28% mentioned fear of movement and 8% identified the misbeliefs embedded in the vignette. Half of the participants linked the patient’s cLBP to multiple factors, yet 38% of those responses were exempt from segments ‘less aligned’ with a biopsychosocial approach.

**Conclusions:**

Physicians generally provided a multifactorial perspective on cLBP, yet relevant factors such as identifying misbeliefs were often overlooked. Approximately, three-quarters did not mention fear of movement, despite evidence supporting this link. Physicians need to be aware of how their perspective can shape potentially harmful or stigmatizing messages, which may contribute to poorer outcomes for patients with cLBP. Given the methodological limitations, further qualitative research is needed to confirm and expand on these findings.

## 1. Introduction

Low back pain (LBP) is the most common musculoskeletal condition prompting consultations with general practitioners (GPs) [1]. Evidence-based guidelines for managing LBP advocate a diagnostic triage to rule out specific underlying spinal pathologies [2–5]. In approximately 90% of cases, no identifiable nociceptive source or pathology can be determined, and LBP is classified as non-specific [2,6]. Once the triage (i.e. specific, nonspecific or radicular LBP) is complete, clinicians are advised to evaluate psychosocial factors, as these are associated with worse outcomes and the persistence of pain [3,5,7–10]. Despite this, research has shown that GPs inadequately assess these psychosocial factors in everyday practice [11], and provide guideline-non-adherent management [12–16]. This gap in adherence is concerning, particularly since 4–20% of patients with LBP will develop chronic low back pain (cLBP) [17], a condition in which the importance of patients’ beliefs, emotions, behaviours, and social or contextual circumstances has been emphasized [3–5,9].

While guideline-adherence is often evaluated through questionnaires or closed-question vignettes (such as Rainville’s vignettes which explore activity and work recommendations [18]), these methods offer limited insight into how physicians conceptualize the causes and influencing factors of cLBP. The International Association for the Study of Pain (IASP) recognizes that biological, psychological and social factors collectively contribute to the pain experience [9], but deeper insights into how GPs perceive and interpret these various influences on patient’s cLBP are rarely explored and represent a critical gap in the current literature.

This study therefore aims to address this gap in the literature by exploring how GPs conceptualize the contributing factors of cLBP and how these perspectives align with evidence-based guidelines. Specifically, the study investigates: (1) physician’s perspectives on the causes and influencing factors of cLBP; (2) the degree to which these perspectives align with a biopsychosocial approach to cLBP management; (3) whether physicians consider multiple factors, including biological, psychological and social, as contributing to the development and persistence of cLBP.

## 2. Materials and methods

This exploratory qualitative study investigated physicians’ responses to an open-ended online survey regarding a clinical vignette depicting a patient with cLBP.

### 2.1. Sampling and recruitment

Dutch- and French-speaking GPs and trainees (i.e. graduated physicians in further training with an interest in musculoskeletal complaints) practicing in Belgium or France were invited to take part in an online survey. A combination of recruitment approaches was used to reach eligible participants [19], including outreach through national organisations (e.g. Domus Medica), academic institutions and regional hospital networks. Invitations were distributed in both Dutch and French, across these professional platforms. To be eligible, participants had to be licensed GPs or trainees involved in primary care, Dutch or French speaking, and actively manage patients with LBP.

### 2.2. Data collection

The survey was available online between August 2021 and March 2023. Respondents accessed the survey in their preferred language via a secure digital platform (Qualtrics; https://qualtrics.com), using any internet-enabled device such as a computer, tablet or smartphone. Prior to participation, the respondents were required to complete and give their consent through a digital informed consent form. As part of this exploratory qualitative study, participants provided basic demographic and professional information, including age, gender, years of clinical experience, and an estimate of the number of new LBP patients managed per month.

The vignette, used in a previous study [20], was developed (in both French and Dutch) by a multidisciplinary team of clinical and academic LBP experts (including a GP, an orthopaedic spine surgeon, and physiotherapists) to reflect a clinically realistic cLBP presentation incorporating multiple biopsychosocial contributing factors, as described in current clinical LBP guidelines [3,5,21,22] (English version attached in Appendix, Supplementary material). The vignette underwent iterative refinement through expert review to ensure: (1) clinical realism and credibility, (2) inclusion of multiple guideline-relevant contributing factors (biomedical, psychological and social) and (3) sufficient ambiguity to elicit diverse physician perspectives without leading responses. External LBP management experts reviewed the final vignette for face validity and clinical appropriateness before deployment [20]. For the thematic analysis, a framework based on the current LBP management guidelines [3,5,21,22] was developed prior to analysing the vignette responses and had been previously applied in a study involving physiotherapists [20]. In total, five relevant themes (factors) were predefined: Beliefs (B), Previous experience with therapy (PE), Emotions (E), Patient’s behaviour (PB) and Contextual factors (CF) [20]. The vignette and thematic framework were pilot tested by the multidisciplinary development team and external LBP experts [20]. The team evaluated whether the vignette reflected the contributing factors outlined in the thematic framework.

After reading the vignette, participants were prompted to respond to the following open-ended question: ‘In your opinion, what are the causes and/or contributing factors to this patient’s pain?’, capturing physicians’ expressed attributions regarding the patient’s cLBP.

### 2.3. Ethical considerations

The study received ethical approval from the Ethics Committee of the Antwerp University Hospital (20/51/714) in February 2021. All participants provided informed consent digitally before continuing to the survey. The study was conducted in accordance with the General Data Protection Regulation. Survey responses were automatically collected and stored via the Qualtrics platform.

### 2.4. Data analysis

A mixed methods analysis of qualitative data was utilized using both a deductive thematic analysis using a flexible framework method and descriptive statistics [23–25]. This approach combines the systematic structure of framework analysis with flexibility to accommodate emergent nuances within the data, making it particularly suitable for analysing responses against established clinical guidelines while remaining open to unexpected patterns.

The research team was already familiar with the framework from prior work [20]. Before commencing formal analysis, the team collectively coded and discussed a pilot sample of 10 Dutch and 10 French responses to refresh their shared understanding of the framework, clarify coding definitions and ensure consistent application across languages.

Step-by-step coding process:

#### 2.4.1. Step 1 – familiarization

Researchers (R.V.; I.D.; N.R.) independently read the complete set of vignette responses in their respective native languages to gain familiarity with the data and note initial impressions. R.V. and I.D. are physiotherapists, lecturers and clinicians specialized in LBP management. R.V. is a doctoral candidate with experience in qualitative research. N.R. is a professor with extensive clinical and research expertise in LBP and pain research. All coders are invested in evidence-based LBP management, which may have influenced interpretation towards guideline concordant perspectives. To address potential bias, researchers worked in close collaboration with an expert team comprising external physiotherapists (internationally recognized LBP experts), a GP, an orthopaedic spine surgeon, and a primary care social scientist specialising in qualitative research and implementation science. This reflexive, collaborative process encouraged critical dialogue, challenged assumptions and supported collective examination of perspectives, strengthening analytical rigor and interpretive credibility [26].

#### 2.4.2. Step 2 – independent coding

Each response was coded sentence-by-sentence. Researchers identified all text segments where physicians expressed attributions about factors contributing to the patient’s chronic pain. Each segment was assigned to one or more of the five predefined themes (Beliefs, Previous Experience, Emotions, Patient Behaviour and Contextual Factors) based on the framework definitions established during pilot testing [20].

#### 2.4.3. Step 3 – directional classification

Within each coded segment, researchers applied a secondary binary classification to assess alignment with guideline recommendations [3,5,21,22]. Segments were classified as either ‘in-line’ (consistent with biopsychosocial guidelines) or ‘less aligned’ (inconsistent with or contradicting guideline recommendations). Classification criteria are detailed in Table 1. This approach is meant to give a description of the directionality of the expressed attributions towards cLBP without implying normative judgement of the physicians’ overall clinical reasoning. For example, statements emphasizing tissue damage or harm (potentially fear-inducing) [5] were classified as ‘less aligned’ with guideline advice to reassure patients [3,5]. Similarly, when the participant invented a diagnosis such as depression (despite the fact that there was no information about a possible depression in the vignette), this was considered a stigma, and therefore a ‘less-aligned’ quote [27]. Conversely, statements acknowledging pain beliefs or known psychosocial risk factors for chronicity [3,5,7,8,21,22,28] were classified as ‘in-line’. When the answer did not provide enough information to consider the segment as either ‘in-line’ or ‘less aligned’, these answers were scored as ‘neutral’ during the analysis. Segments that were deemed plausible (such as emotions related to grief), but that were not explicitly mentioned in the vignette, were scored neutral as well. Regarding the theme ‘Beliefs’, answers were further subdivided into three ‘in-line’ subthemes (B1, B2, B3, Table 1) and three ‘less aligned’ subthemes (B4, B5, B6, Table 1) with their own descriptions. When a response was given that could not be classified in any of the aforementioned themes, they were categorized in ‘Other’.

**Table 1.** Relevant themes and their description, contributing to cLBP in the clinical vignette.

Theme	Category	Code	Description
Beliefs	In-line	B1	Recognizing the misbelief that ‘the patient’s cLBP is still caused by tissue damage or by a biomechanical cause’.
		B2	Recognizing the misbelief that ‘rest/avoiding movement is necessary to decrease the patient’s cLBP’.
		B3	Recognizing the misbelief that ‘moving in a very specific way is necessary to decrease the patient’s cLBP’.
	Neutral	B-n	The information in the answer was not detailed enough to correctly interpret the participants answer. Or the participant gave plausible answer but that was not explicitly mentioned in the vignette.
	Less aligned	B4	The participant attributes the cause of the patient’s cLBP to ‘bad movements’ or ‘postures’, etc. (which can be considered as implying or emphasizing a harmful message).
		B5	The participant explains that ‘ageing’ is the cause of the patient’s cLBP.
		B6	The participant attributes ‘a specific pathology or impairment in anatomical structure’ as cause of the patient’s cLBP.
Previous experience	In-line	PE1	The participant recognizes ‘the patient’s previous unsuccessful experience is associated with the patient’s cLBP’ (because it was focussed on harm and danger with wordings such as ‘keep his vertebrae in place’, ‘move in a specific way to prevent damage’, etc.).
	Neutral	PE-n	The information in the answer was not detailed enough to correctly interpret the participants answer. Or the participant gave plausible answer but that was not explicitly mentioned in the vignette.
	Less aligned	PE2	The participant mentions that ‘the patient’s previous experience was unsuccessful because the previous treatment was executed not well enough’ (i.e. emphasizing the harmful messages).
Emotions	In-line	E1	The participant recognises the fact that ‘the patient’s worries about pain and movements might contribute to the patient’s cLBP’.
	Neutral	E-n	The information in the answer was not detailed enough to correctly interpret the participants answer. Or the participant gave plausible answer but that was not explicitly mentioned in the vignette.
	Less aligned	E2	The participant concludes that ‘depression’ or ‘a mentally fragile situation’ is the cause of the patients’ cLBP (i.e. stigmatizing the person, as depression was not mentioned in the vignette).
Patients behaviour	In-line	PB1	The participant recognises the ‘sedentary aspect or the avoidance behaviour of the patient as a contributor to the patient’s cLBP’.
	Neutral	PB-n	The information in the answer was not detailed enough to correctly interpret the participants answer. Or the participant gave plausible answer but that was not explicitly mentioned in the vignette.
	Less aligned	PB2	The participant states that the ‘patient suffers from cLBP because he did his exercises wrong’.
Contextual factor	In-line	CF1	The participant recognises that ‘unhelpful influence of the family may contribute to the patient’s cLBP’.
	Neutral	CF-n	The information in the answer was not detailed enough to correctly interpret the participants answer. Or the participant gave plausible answer but that was not explicitly mentioned in the vignette.
	Less aligned	CF2	The participants concludes that ‘the patient is not able to adapt to his changed environment’ (although no information was given in the vignette).
Other		O	The given answer, or part of it, could not be classified in the aforementioned themes.

cLBP: chronic non-specific low back pain; B: beliefs; PE: previous experiences; E: emotions; PB: patient behaviour; CF: contextual factors; O: other; …-n: neutral; description: guiding text for the researchers to help classify the content of the answers in their respective (sub)themes.

#### 2.4.4. Step 4 – consensus meetings

Following independent coding, paired researchers (Dutch: R.V. and I.D.; French: N.R. and I.D.) met to compare coding line-by-line. Discrepancies in theme assignment or directional classification were discussed until consensus was reached through negotiated agreement. When consensus could not be achieved, the issue was discussed in the wider team. Inter-coder agreement was high: 91% for Dutch responses and 90% for French responses, calculated as (total coded segments – segments requiring consensus discussion)/total coded segments.

#### 2.4.5. Step 5 – pattern identification

Once all responses were coded and consensus achieved, researchers examined patterns across the dataset: which themes were most frequently addressed, which were overlooked, and the distribution of ‘in-line’ versus ‘less aligned’ attributions within and across themes.

While the framework provided structure through predefined themes, the ‘flexible’ aspect allowed coders to: (1) assign multiple themes to single segments when physicians discussed interconnected factors, (2) capture nuances in how physicians expressed attributions (e.g. tentative vs. definitive statements) and (3) note emergent patterns not anticipated by the framework, which informed our interpretation of findings.

For all the themes, the frequency of occurrence of coded segments was counted in the responses. Additionally, an inductive analysis method was adopted in the form of a reflective analysis. This allowed us to further explore the content of the answers. (i.e. combination of ‘in-line’ and ‘less aligned’ approaches) within a theme containing multiple ‘less aligned’ quotes.

Finally, as the IASP recognizes pain as a personal experience that is influenced by multiple factors [9], the ‘multifactorial’ dimension of pain was assessed. When physicians only used one or two themes in their perspective on the cause and/or contributing factors to cLBP, this was considered as ‘few-factorial’. The team agreed that the few- and multifactorial answers should be screened for the presence of ‘less aligned’ quotes as the presence of even one ‘less aligned’ quote could leave conflicting messages (e.g. recognising that the patient is sedentary and needs to be more active, but simultaneously stating the muscles of the back are too weak and are responsible for the cLBP) and have an enduring impact on the patient [10]. Therefore, answers categorised as either ‘few factorial’ or ‘multifactorial’ were descriptively analysed on whether or not they included a ‘less aligned’ quote.

## 3. Results

### 3.1. Descriptive data

In total, 76 physicians (62% female, 38% male) completed the survey. Amongst those physicians, 34% were trainees, and 66% were GPs, representing a median work experience of 10.5 years. Forty-nine percent of the physicians saw 6–15 new LBP patients each month. Additional details are listed in Table 2.

**Table 2.** Descriptive data.

	*n* (%)	Median (IQR)
Population		
Trainees	26 (34%)	
General practitioners	50 (66%)	
Gender		
Female	47 (62%)	
Male	29 (38%)	
Estimation number of new LBP patients/month		
1–5	3 (4%)	
6–10	24 (32%)	
11–15	13 (17%)	
16–20	6 (8%)	
>20	4 (5%)	
Age (years)		31.5 (12.25)
Work experience (years)		10.5 (17.75)

*n*: amount of participants; IQR: interquartile range; LBP: low back pain.

### 3.2. What are the physicians’ perspectives on contributing factors to cLBP

**Figure 1.**
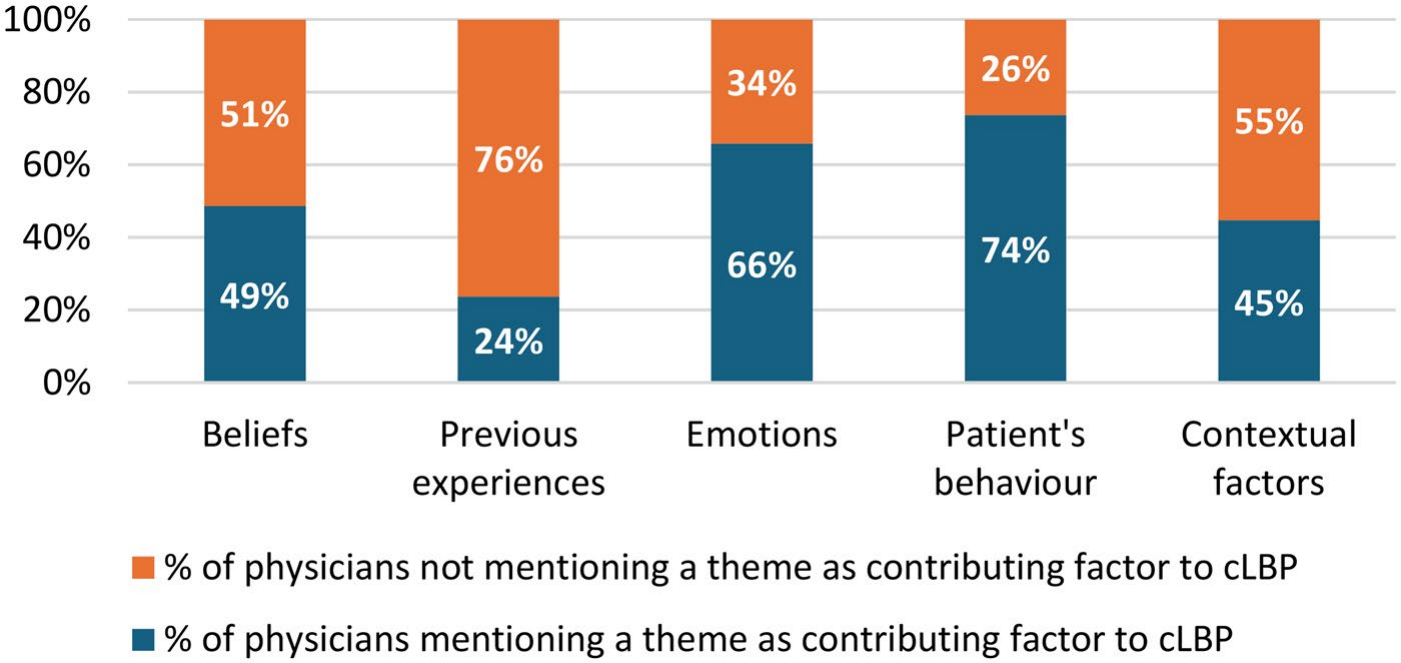
Frequency of themes.

Physicians used very brief explanations when reporting contributing factors to cLBP with a median of 11 words (IQR: 11, maximum: 43). Physicians mainly mentioned ‘Patient’s behaviour’ (74%) and ‘Emotions’ (66%) as contributing factors towards cLBP (Figure 1). Half of the participants mentioned ‘Beliefs’ (49%) and ‘Contextual factors’ (45%) while ‘Previous experiences’ in relation to pain was barely considered (24%) (Figure 1).

### 3.3. Exploration of physician’s perspectives on cLBP

Table 3 presents the proportion of physicians’ quotes categorised according to predefined (sub)themes, alongside examples of quotes. A majority of physicians (71%) identified the patient’s sedentary lifestyle (PB1) as a relevant contributing factor. Only 28% recognized fear of movement (E1) as a contributor to the patient’s cLBP. Other ‘in-line’ themes were mentioned infrequently, including patient’s misbeliefs (B1–3: <10%), previous negative experiences with therapy (PE1: 5%), and family advice to avoid further damage (CF1: 9%). Physicians more commonly referred to the patient’s psychosocial context, such as the passing of his wife, mentioned in the context of ‘grieving’ (E-n: 42%) or ‘living alone’ (CF-n: 42%), but the psychosocial context related to the passing of his wife was not included in the vignette.

**Table 3.** Content and proportions of quoted themes.

Theme	Category	Code	*N*%	Example of quotes
Beliefs	In-line	B1	5%	‘The misbelief that the back is vulnerable’.
		B2	8%	‘The misbelief that he has to rest because of his pain’.
		B3	5%	‘The misbelief that it is necessary to avoid movement in order to not have pain’.
	Neutral	B-n	3%	‘Load his back during cleaning of his car’.
	Less aligned	B4	29%	‘Weak muscles’; ‘Wrong posture’
		B5	5%	‘Age’
		B6	12%	‘Discal hernia’; ‘Osteoarthritis’
Previous experience	In-line	PE1	5%	‘The patient did not receive the recommendation to keep moving’.
	Neutral	PE-n	12%	‘No improvement despite following the prescriptions of the general practitioner and physiotherapist’.
	Less aligned	PE2	8%	‘Lack of acute analgesic treatment’.
Emotions	In-line	E1	28%	‘Fear of movement’; ‘Anxiety of the patient’.
	Neutral	E-n	42%	‘Grief’; ‘Loneliness’
	Less aligned	E2	5%	‘Depression’
Patients behaviour	In-line	PB1	71%	‘Sedentary lifestyle’; ‘Physical deconditioning’; ‘Lack of physical activity’
	Neutral	PB-n	1%	‘Change of activities’.
	Less aligned	PB2	0%	–
Contextual factor	In-line	CF1	9%	‘His children are advising him to rest’.
	Neutral	CF-n	42%	‘Living alone’; ‘The death of his wife’.
	Less aligned	CF2	0%	–
Other		O	18%	‘Chronic pain’; ‘Non-specific pain’

*N*%: percentage of the total population having a quote categorised in a (sub)theme; B: beliefs; PE: previous experiences; E: emotions; PB: patient behaviour; CF: contextual factors; O: other; …-n: neutral.

A subset of responses included ‘less aligned’ quotes. These included the stigmatizing suggestion that the patient was suffering from depression (E2: 5%) or mentioned that he had not received adequate acute analgesic treatment (PE2: 8%). Furthermore, 29% of physicians provided misbeliefs of their own (B4) as they considered the patient’s weak muscles or wrong posture to be an important cause of his cLBP. Similarly, 12% of the physicians suspected a specific cause or impairment in anatomy to be linked to the patient’s cLBP (B6) and 5% indicated the patient’s ‘Age’ (B5) as relevant. Finally, 18% of physicians referred to the terms ‘chronic pain’ or ‘non-specific pain’ (O), without further elaboration, as a contributing factor to the clinical presentation.

### 3.4. Reflective analysis

**Figure 2.**
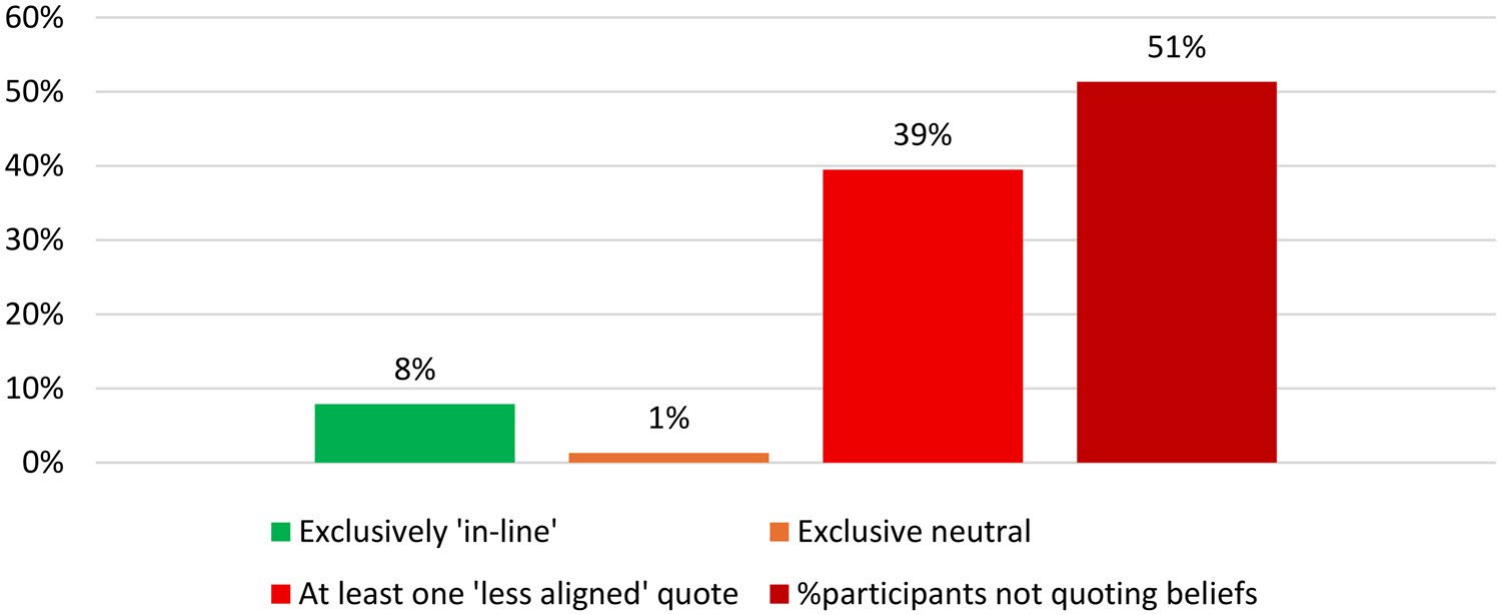
Reflective analysis – theme beliefs.

Compared to the other themes, the theme ‘Beliefs’ showed a substantial number of answers categorised as ‘less aligned’. When exploring the beliefs associated with pain, only 8% of the physicians identified the misbeliefs present in the vignette and linked them to the patient’s pain (Figure 2). Thirty-nine percent (Figure 2))of the physicians introduced at least one misbelief of their own (B4–6, Table 3) and 51% did not report anything at all about the present pain beliefs (Figure 2). Although 71% of physicians (PB1, Table 3) recognized the patient’s sedentary lifestyle, only 7% (*n* = 5) physicians did so while also (i.e. remaining exclusively ‘in-line’) acknowledging the misbeliefs presented in the vignette (B1–3, Table 3).

### 3.5. Use of multiple factors when considering contributing factors to chronic pain

**Figure 3.**
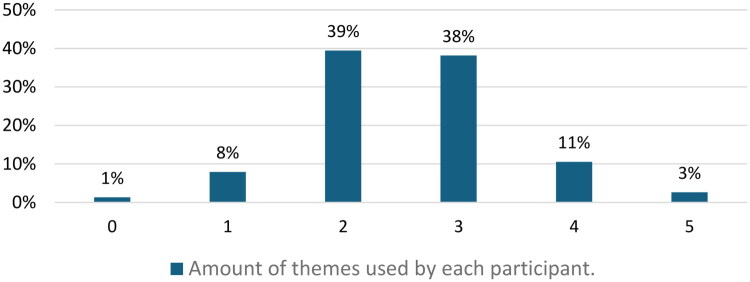
Use of multiple factors when explaining contributing factors to chronic pain.

As pain is linked to multiple factors, the number of themes that were included in the explanation of the physicians was counted. Up to 48% of the physicians considered pain to be ‘few factorial’ (i.e. they did not mention more than two different contributing themes when explaining the patient’s pain). Amongst the participants with a ‘multifactorial’ approach (52%), 38% used three different themes and 14% considered four to all five predefined themes as contributing factors in the fictive cLBP case (Figure 3). Interestingly, only 38% of physicians who provided a ‘multifactorial’ response did so without including any ‘less aligned’ quotes. Meanwhile, 70% of ‘few factorial’ responses consisted exclusively of ‘in line’ quotes. One percent gave an answer that could only be categorised as ‘Other’ (i.e. a quote not fitting the predefined themes) and were not assigned in any of the five predefined themes.

## 4. Discussion

This study is the first to explore how physicians conceptualise cLBP by capturing their expressed attributions regarding contributing factors within a clinical vignette context. While many physicians recognized the potential role of sedentary behaviour and emotional/contextual influences, only 28% mentioned fear of movement and a small minority (8%) identified the presence of misbeliefs as contributing to the patients’ cLBP, despite the central role these misbeliefs play in the persistence of pain.

### 4.1. Framing the expressed attributions within a biopsychosocial approach of LBP management

Although most responses reflected a multifactorial perspective of cLBP, the content of the answers aligned with established biopsychosocial guidelines in only 38% of the participants. This suggests that while physicians are aware of the complex interplay of factors in cLBP, key psychosocial risk factors, particularly *misbeliefs related to pain or movement*, are often overlooked. This finding is concerning, as unhelpful beliefs about LBP are well-known risk factors for chronicity and disability [3,5,7–9,21,22,28]. Persistent misbeliefs can even perpetuate the false notion of spinal vulnerability, ultimately driving patients towards activity avoidance and poorer outcomes [8,10,16,28–31]. Identifying and addressing these misbeliefs is a cornerstone of evidence-based management approaches, and could help them to reassure patients with non-specific LBP [3,5,7,8,21,22].

A majority of physicians identified *sedentary behaviour* as a contributing factor, reflecting an awareness of its association with an increased risk of cLBP [32,33]. While this might be encouraging at first sight, only 7% of physicians associated this with the many misbeliefs in the vignette (i.e. ‘rest or avoiding movement is necessary to decrease the patient’s cLBP’, ‘the patient’s cLBP is still caused by tissue damage or a biomechanical cause’, ‘moving in a specific way is necessary to decrease the patient’s cLBP’), and approximately one-third demonstrated similar misbeliefs themselves. Without simultaneously addressing underlying misbeliefs, identifying sedentary behaviour may have limited impact. Advising patients to stay active, without effectively challenging misunderstandings about pain, is unlikely to result in meaningful behavioural change.

*Emotional and psychological factors* were acknowledged by around two-thirds of GPs, but specific issues such as fear of movement or pain related worry were explicitly identified in fewer than one-third responses. This is surprising given the well-established role of fear-avoidance in the development and persistence of cLBP [28,29,34] and influences patients towards a negative outcome [10,28]. Interestingly, nearly half of the physicians mentioned the recent death of the spouse as a possible contributor in the form of ‘grief’ or ‘living alone’, while nothing about how the death of his wife affected his emotions or contextual situation was included in the vignette. While grief may indeed play a role, this tendency to infer psychosocial stressors while overlooking more immediate pain-related beliefs and behaviours suggests a potential misalignment in how physicians interpret and prioritise psychosocial information. A further concern is that some physicians attributed the patient’s cLBP primarily to ‘depression’, despite the presence of multiple other relevant factors in the vignette. Such focus risks stigmatization and may alienate patients, especially when not grounded in a comprehensive assessment of the patient’s experience [27].

Few physicians mentioned the *patient’s previous (unsuccessful) therapeutic experiences*, despite evidence linking prior negative treatment encounters with worse prognoses [35]. GPs rarely acknowledged the patient’s *social context* (overprotection of the family, encouraging a sedentary behaviour). This is a missed opportunity, as engaging family in discussions around pain and activity has been shown to support favourable outcomes [36].

Some responses categorized under ‘*other*’ suggested that ‘non-specific pain’ or ‘chronic pain’ were causes of the patient’s symptoms, an interpretation that lacks clinical coherence. These responses underscore the need for future research to explore physicians’ conceptual understanding of cLBP and its contributing factors.

Overall, responses were typically *brief and lacked the depth* necessary to reflect a thorough biopsychosocial approach. Yet for patients with LBP, consistent, clear and personalised communication is essential for providing reassurance and supporting active management strategies [37]. This reassurance is the first, necessary, step in the management of the patient [3,5,21,22]. Only once patients feel reassured and understood can GPs begin to encourage meaningful behavioural changes, such as increasing physical activity and re-engagement in care and daily life. It was not within the scope of this study to analyse the association between word count and the amount of themes or the likelihood of ‘in-line’ coding. However, it is reasonable to assume that participants with a higher word count would present a higher number of themes. Interestingly, our findings indicated that few ‘multifactorial’ responses were fully ‘in-line’ with a biopsychosocial approach within the context of the vignette, whereas the opposite was true for ‘few factorial’ responses. This suggests that a higher theme count does not necessarily equate to providing ‘in-line’ expressions. Future research should employ in-depth interviewing techniques to allow physicians to elaborate on their perspectives regarding contributing factors to cLBP, in order to explore whether their responses genuinely reflect a thorough biopsychosocial approach. This should be complemented by observations of actual patient-physician interactions to determine whether this thorough biopsychosocial perspective is implemented in daily practice.

### 4.2. Contextualising the findings within existing barriers to LBP guideline implementation

The findings of this study align with previous research exploring barriers physicians experience in adhering to the clinical LBP guidelines [16,38–41]. A systematic review of qualitative studies found moderate evidence that physicians commonly provide inappropriate advice on rest and activity, and are unsure about how and when exercise is beneficial [38]. This includes providing mixed messages about activity levels to patients (e.g. ‘being protective and active at the same time’) [38,42], which is consistent with the considerations made in the current study. While most physicians recognised sedentary behaviour as a contributing factor, other relevant factors were often overlooked (e.g. misbeliefs related to pain, patient’s fear of movement). Some may even have inadvertently reinforced these misbeliefs through ‘less aligned’ responses known to perpetuate fear and avoidance behaviours in LBP patients, such as segments referencing ‘weak muscles’ and ‘wrong posture’, potentially leaving an enduring impact on the patient’s pain behaviour and perceptions [10,39,43,44]. This is consistent with research identifying misconceptions amongst physicians, including beliefs that ‘unsafe postures’ cause LBP [16]. GPs may reflect their own fear-avoidance beliefs onto their LBP management [42,45]. Research has demonstrated that it is not possible to identify a specific nociceptive source in people with non-specific LBP, particularly in chronic cases [8,46,47]. Yet physicians report diagnostic uncertainty in distinguishing specific from non-specific LBP [38,41,42,48], and often struggle to reassure patients that nothing is structurally wrong with their back or to explain why imaging is not required [38]. This uncertainty also appears to extend to the broader multifactorial influences on pain [9], as uncertainty in LBP care is common when clinicians consider patients’ personal, social and contextual factors [40]. This is consistent with physicians in the present study seldomly attributing relevance to the contextual, emotional (i.e. fear or worry) and ‘previous experiences’ presented in the vignette. These feelings of uncertainty are reported to be often managed by avoiding the topic or providing narrow responses [40,41]. This may align with the physicians’ brief responses in the present study. However, future qualitative interviews should explore this uncertainty and whether it influences physicians’ conceptualisation of cLBP.

The views expressed by physicians in this study may indicate that a multifactorial perspective of cLBP, as recommended in clinical guidelines, is not yet fully adopted in clinical practice. This is consistent with research indicating that clinicians experience challenges in effectively implementing a multifactorial biopsychosocial approach for managing pelvic girdle pain [49]. Furthermore, previous studies have highlighted the need to adopt such multifactorial approaches in LBP management and have developed clinical reasoning frameworks to support this, specifically because clinicians have difficulties with integrating these approaches into their practice [50,51]. Research on guideline implementation also reported that clinicians demonstrate low adherence to published guidelines and some perceive them as restrictive [52,53]. Instead, they tend to rely on their clinical experience and judgement, including accepting practices amongst peers, rather than the guidance provided in clinical guidelines [52,53]. However, the extent to which physicians hold multifactorial perspective and how their perceptions of guidelines influence their decision-making warrant further exploration. Future research should verify these interpretations in actual clinical context using methods such as simulated-patients, video-recordings and interviews, which would allow a more in-depth understanding of physicians’ interpretations of the multifactorial nature of (chronic) LBP. If confirmed, these perspectives could limit physicians’ ability to deliver consistent, guideline-based messages. Addressing these challenges is essential, as effective communication about the multifactorial nature of pain [9] and the importance of maintaining activity [3,22] are central to improving patient outcomes.

### 4.3. Strengths and limitations

This study is amongst the first to explore the physicians’ perspectives on how they identify and integrate biopsychosocial factors when considering cause and influencing factors of cLBP. By including both GPs and trainees, this study captured the perspectives of a younger physician population, providing insight into both current and future GPs. However, several limitations must be considered. First, the brevity of participants’ responses, likely influenced by the online survey format, limited the depth of analysis and necessitated classifications into neutral categories for ambiguous answers. Furthermore, the coding framework also contains potential interpretation bias as it was constructed in what the team found relevant within the clinical vignette. However, to minimize this bias, a multidisciplinary team consisting of internal and invited external LBP experts reviewed the development of both the clinical vignette and framework to ensure its completeness and consistency towards the clinical guidelines and compared it with the clinical guidelines. Second, while the clinical vignette was designed to reflect the multifactorial nature of cLBP and align with guideline recommendations, its succinct format may have influenced the scope of participants’ responses. An in-depth interview might have provided more elaborate responses. Future studies should further explore the clinical reasoning of GPs in primary care settings, potentially through interviews and video analysis of physician–patient interactions. Third, the generalisability of findings is limited by the modest sample sizes of both GPs and trainees. While all participants had clinical experience with LBP patients, trainees were still in supervised training and may have less independent clinical experience than established GPs. This study did not make subgroup comparisons between GPs and trainees, as our exploratory qualitative design aimed to capture the range of conceptualisations present across primary care physicians rather than to quantitatively compare groups. Nevertheless, the inclusion of trainees provides valuable insight into how emerging GPs currently conceptualise cLBP, with important implications for medical education and training. Finally, recruitment bias cannot be excluded as the study relied on voluntary participation through professional organizations and demographic information about non-responders was unavailable.

### 4.4. Implications for practice

This study highlights the opportunity to enhance GPs’ training in addressing unhelpful beliefs and biopsychosocial factors in cLBP management. By prioritizing the identification and correction of misbeliefs, physicians can help patients better understand the multifactorial nature of pain. Clear, consistent and personalized communication, aligned with biopsychosocial guidelines, will empower GPs to deliver more effective care and foster greater patient engagement in active management.

### 4.5. Future research

This paper provides new insights into what direction physicians’ conceptualise cLBP. However, considering the methodological limitations of this study, future research should explore in more depth the clinical reasoning processes of GPs. Through interviews, physicians will have the opportunity to explicitly share their conceptualisation of cLBP. Future studies should analyse what kind of information GPs identify as a relevant contributing factor, and which factors are left out. Interviews should explore physicians’ argumentation and investigate in what degree diagnostic uncertainty played a role in their decision-making. This should be combined with analysing the physician–patient in daily practice using video recordings and simulated patients to observe whether their communication matches their clinical reasoning. Depending on these results, specific educational interventions can be aimed at improving physicians’ clinical reasoning and implementation of the clinical guidelines to stimulate a multifactorial conceptualisation of cLBP in line with the clinical LBP guidelines.

Future research could meaningfully extend this work by examining how physicians’ conceptualisations differ across a range of clinical presentations, such as varying degrees of chronicity, pain presentation, complexity and patient demographics.

## 5. Conclusions

This study is the first to explore how physicians conceptualise cLBP and provides new insights into what direction physicians attribute contributing factors towards the patient’s pain. The findings of this study lay the groundwork for future in-depth qualitative research on how physicians conceptualise cLBP in combination with physicians–patient interaction in daily practice. Although the current research has its methodological limitations, this study provides new insights that can help physicians in addressing unhelpful beliefs, integrating a multifactorial perspective, and delivering consistent, evidence-based messages potentially helping physicians to align with clinical guidelines, ultimately improving patient outcomes and promoting active management strategies.

## Supplementary Material

Supplemental Material

## Appendix

### Fictive clinical vignette depicting a patient with chronic non-specific low back pain

**Table:**

*A 68-year-old man comes to your practice. He complains of persistent low back pain (VAS score of 6/10). The pain is located at level L4/L5. During certain activities, the pain intensity can increase and in that moment the pain also radiates to the right buttock. In his life, he has never really practiced any physical activity. The man lives alone. His wife died of cancer last year.*
*This pain started a year ago after he had cleaned his car with a vacuum cleaner. The pain was very intense at that moment. His children advised him to rest so he would avoid further damage to his back. After a few days, the intense pain was still present. As he was really worried, he decided to consult his general practitioner. The general practitioner advised him not to worry and said that the pain would go away if he got enough rest.*
*However, the pain did not improve much in the following weeks. He agreed with his general practitioner to see a physiotherapist. The latter explained that he needed to strengthen his back and abdominal muscles to keep his vertebrae in place. The physiotherapist also showed him how to correctly bend forward to prevent damage to his back and told him which movements to avoid.*
*Despite following the advice of the physiotherapist, the pain persists. His general practitioner prescribed physiotherapy again. Since the previous physiotherapy sessions did not lead to any improvement, he now consults you to have his back examined and treated.*
