# Supplementary material for: Exploring physicians’ perspectives on influencing factors of chronic non-specific low back pain: a qualitative study
Source: Scand J Prim Health Care. 2026 Mar 16;44(1):2640406. doi: 10.1080/02813432.2026.2640406 (PMC12997369; doi:10.1080/02813432.2026.2640406)
Supplement: Supplemental Material [file IPRI_A_2640406_SM8562.docx]

**Appendix**

| **Fictive clinical vignette depicting a patient with chronic non-specific low back pain.** |
| --- |
| *A 68-year-old man comes to your practice. He complains of persistent low back pain (VAS score of 6/10). The pain is located at level L4/L5. During certain activities, the pain intensity can increase and in that moment the pain also radiates to the right buttock. In his life, he has never really practiced any physical activity. The man lives alone. His wife died of cancer last year.*  *This pain started a year ago after he had cleaned his car with a vacuum cleaner. The pain was very intense at that moment. His children advised him to rest so he would avoid further damage to his back. After a few days, the intense pain was still present. As he was really worried, he decided to consult his general practitioner. The general practitioner advised him not to worry and said that the pain would go away if he got enough rest.*  *However, the pain did not improve much in the following weeks. He agreed with his general practitioner to see a physiotherapist. The latter explained that he needed to strengthen his back and abdominal muscles to keep his vertebrae in place. The physiotherapist also showed him how to correctly bend forward to prevent damage to his back and told him which movements to avoid.*  *Despite following the advice of the physiotherapist, the pain persists. His general practitioner prescribed physiotherapy again. Since the previous physiotherapy sessions did not lead to any improvement, he now consults you to have his back examined and treated.* |
